# Care for post-stroke patients at Malaysian public health centres: self-reported practices of family medicine specialists

**DOI:** 10.1186/1471-2296-15-40

**Published:** 2014-03-02

**Authors:** Aznida F Abdul Aziz, Nor Azlin Mohd Nordin, Noor Abd Aziz, Suhazeli Abdullah, Saperi Sulong, Syed M Aljunid

**Affiliations:** 1 United Nations University International Institute for Global Health, Kuala Lumpur, Malaysia; 2 Department of Family Medicine, Faculty of Medicine, Universiti Kebangsaan Malaysia Medical Centre, Jalan Yaacob Latif, Bandar Tun Razak, 56000 Cheras, Kuala Lumpur, Malaysia; 3 School of Rehabilitation Sciences, Faculty of Health Sciences, Universiti Kebangsaan Malaysia, Kuala Lumpur, Malaysia; 4 Klinik Kesihatan Marang, Terengganu, Malaysia; 5 Department of Health Information, Universiti Kebangsaan Malaysia Medical Centre, Kuala Lumpur, Malaysia; 6 International Casemix & Clinical Coding Centre, Universiti Kebangsaan Malaysia, Kuala Lumpur, Malaysia

**Keywords:** Post stroke, Primary care, Healthcare delivery

## Abstract

**Background:**

Provision of post stroke care in developing countries is hampered by discoordination of services and limited access to specialised care. Albeit shortcomings, primary care continues to provide post-stroke services in less than favourable circumstances. This paper aimed to review provision of post-stroke care and related problems among Family Medicine Specialists managing public primary health care services.

**Methods:**

A semi-structured questionnaire was distributed to 121 Family Physicians servicing public funded health centres in a pilot survey focused on improving post stroke care provision at community level. The questionnaire assessed respondents background and practice details i.e. estimated stroke care burden, current service provision and opinion on service improvement. Means and frequencies described quantitative data. For qualitative data, constant comparison method was used until saturation of themes was reached.

**Results:**

Response rate of 48.8% was obtained. For every 100 patients seen at public healthcentres each month, 2 patients have stroke. Median number of stroke patients seen per month is 5 (IQR 2-10). 57.6% of respondents estimated total stroke patients treated per year at each centre was less than 40 patients. 72.4% lacked a standard care plan although 96.6% agreed one was needed. Patients seen were: discharged from tertiary care (88.1%), shared care plan with specialists (67.8%) and patients who developed stroke during follow up at primary care (64.4%). Follow-ups were done at 8-12 weekly intervals (60.3%) with 3.4% on ‘as needed’ basis. Referrals ranked in order of frequency were to physiotherapy services, dietitian and speech and language pathologists in public facilities. The FMS’ perceived 4 important ‘needs’ in managing stroke patients at primary care level; access to rehabilitation services, coordinated care between tertiary centres and primary care using multidisciplinary care approach, a standardized guideline and family and caregiver support.

**Conclusions:**

Post discharge stroke care guidelines and access to rehabilitation services at primary care is needed for post stroke patients residing at home in the community.

## Background

Primary care physicians or family physicians or known as Family Medicine Specialists (FMS) in Malaysia, play a major role in ensuring continuity of care for patients with chronic non-communicable diseases in the community. The four-year postgraduate FMS training in this country trains medical officers in providing specialist-grade services at primary care level.

In line with National Strategic Plan for the management of non communicable diseases (NSPNCD) [1], there is now a greater emphasis on optimising the provision of health management across stakeholders, promoting shared care management of these conditions between hospital care and primary care specialists [1]. Although stroke has been listed as one of the disease priorities for National Reduction in NCD prevalence, progress on this field especially after post hospital transfer is much less compared to chronic kidney disease or diabetes [2,3]. The shared care concept is not replicated in the care of post stroke or long-term stroke patients in the community. The NSPNCD acknowledges that within the public health sector, the care delivery becomes disjointed when it involves intersectoral collaboration. Hence the 9th Malaysian Plan (Health section) (2006-2010) identifies the need to consolidate the current services to achieve better health for all as a priority [4]. Intensifying resources and services by fortifying multidisciplinary care for post stroke patients is one area.

Acute care for stroke in Malaysia is mainly provided in tertiary hospitals, where patients have direct access or via referral from primary care, depending on the circumstances at presentation. Patients presenting with acute symptoms are at liberty to seek care from any public or private health facility. Secondary care provision usually involves rehabilitative services, which are provided either during admission, or as outpatient basis once the patient is discharged home. The average length of stay for an acute stroke admission is 7.48 days according to Hamidon et al (2003). Access to secondary care is usually initiated by the specialists caring for the patient during the acute phase or by the primary care team when the patients seeks post stroke care once they return to their own homes.

Traditionally, the burden of caring for stroke patients falls on immediate family members once they are discharged from hospital. These patients often end up seeking further treatment from primary care facilities (i.e. healthcentres) and to date, the burden of caring for stroke patients in the community in Malaysia is unknown. There is a lack of local data on incidence and prevalence of stroke in this country, although it has been reported by the Ministry of Health that cerebrovascular disease/stroke is the 3rd among 8 leading causes of mortality and morbidity in Malaysia [5].

Local studies are mainly limited to acute stroke incidences and treatment during acute phase at tertiary level [6]. Prospective trials assessing long term outcomes of stroke patients treated at community level are scarce. There are also an unaccountable proportion of patients who develop stroke and receive treatment at primary care level without ever being admitted to tertiary or secondary hospitals.

**Figure 1:**
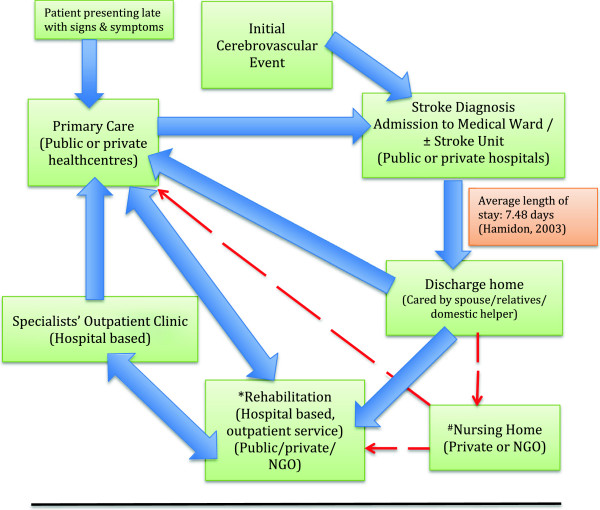
**Post stroke care service provision in Malaysian health facilites-public and private sector.** *Stroke or neurorehabilitation services are limited to tertiary & some private hospitals. ^#^Services & facilities available at private Nursing Homes vary, according to charges.

Post stroke care for patients discharged home after acute stroke is provided either at Specialist Clinics (a.k.a. Klinik Pakar Perubatan) based in tertiary or state referral centres and secondary/district hospitals or at outpatient primary care facilities. The latter can range from hospital-based Outpatient Units or public healthcentres (a.k.a ‘Klinik Kesihatan’) or even private general practitioner clinics. At public healthcentres, FMS are expected to lead the primary care team to provide ongoing care for patients who have been discharged from tertiary care. Thus, making the post stroke care spectrum in Malaysian public health facilities being provided mainly either between tertiary or secondary care directly to primary care. The exact proportions of care undertaken by these care providers is unknown. (Please refer to Figure 1). Similar services are also provided by the private healthcare service providers in which the patients self-finance with or without using health insurance schemes.

Post discharge stroke care only gained recognition in the last two decades, by which concepts of early supported discharge, further rehabilitation and follow-up care were discussed and highlighted. However the deliverance of care in the community is far from ideal due to factors such as variations in health care delivery systems, problem of access to specialised stroke care and lack of guidelines on long-term stroke care at community level are some examples. Gaps exist in the transfer of care from tertiary to primary care although it is expected that the latter would be in the best position to provide continuity of care for this chronic NCD.

Realizing these gaps, we aimed to develop an integrated care pathway for the management of post stroke patients in the community. The first phase of the development of the pathway was to obtain background information from the main service providers in the community, i.e. the primary care physicians regarding the estimated clinical burden of stroke patients seen in their practice, services provided, knowledge on post stroke care provision, problems faced in delivery of care and their expectations for improvement of service at public healthcare facilities.

In general, it is hoped that the findings of this study will identify problems encountered by the primary care physicians in delivery of care to post stroke patients in the community. Specifically, the gaps in service provision will be identified and targeted to enable delivery of structured stroke care management at primary care level in a seamless manner. The findings of this study will aid us in designing a care pathway for post stroke patients which aims to achieve better health through consolidation of services.

## Methods

### Study design

This was a cross sectional survey done to review the services provided for post stroke patients as well as to estimate the burden of stroke care at public primary care facilities. The family medicine specialists were asked to recall their experiences on provision of care for patients who had stroke as a diagnosis in the past one year at their practice.

### Study setting and time scale

The survey was part of a bigger study on integrated care pathway conducted at United Nations University International Institute for Global Health, Kuala Lumpur. This part of the study took place between June 2011 and February 2012.

### Participants

Study population was FMS managing health services at government primary health care centres. Potential participants were identified by reviewing database of public health institutions and FMS registry maintained by the Ministry of Health, Malaysia and the Malaysian Family Physician Association, respectively. Altogether, there were 176 registered FMS serving the public primary healthcare centres throughout Malaysia at the time the study was conducted.

Calculation of sample size was based on guideline for planning a survey by Kretcjie and Morgan (1970) [7]. Allowing for 5% margin of error, with 95% confidence interval and population proportion of 0.5 based on an assumption that almost half of the FMS would have managed a stroke patient or stroke related complications in their practice, the calculated size of sample required for the survey was 121. Estimating a 50% dropout rate, all 176 FMS were approached for the survey.

Approval for the study was obtained from the UKM Ethics and Research Committee (Research ID: UKM-GUP-2011-327) & National Medical Ethics & Research Committee (MREC) (Research ID: NMRR-11-1074-10358).

### The questionnaire

The 77-item questionnaire comprises 13 items on current service provision for stroke, 10 items on knowledge on management of patients after stroke, 15 items on medical complications, 15 items functional status and activities of daily living (adapted from Barthel Index and Brady & Lawton’s IADL scales), 13 items on comprehensive stroke care, 6 items on expectations for improvement of care, 5 items on Stroke carer assessment (adapted from Carer Strain Index).

The last section was an open-ended questionnaire, which asked respondents to write their views on “*What is the one important aspect of stroke management that you think is important in your practice in primary care*?”

This paper will focus on macro aspects of service provision and areas for improvement although the questionnaire covered an extensive area on post stroke care provision. The remaining sections of the survey will be discussed in another paper focussing on the management of post stroke complications at community level.

Two experts who manage stroke patients at community level did the content validation on the close-ended questions. Pilot testing was done on Final year Family Medicine postgraduate trainees prior to distribution. Modifications were made to the question on ‘problems encountered in transfer of care process’ based on feedback received during the pilot testing, i.e. to rank response options according to order of frequency, from most common [1] to least common [8] encountered problem. The responses from the pilot study were not included in the final analysis.

The time required for completion of questionnaire averages 15 minutes. Enquiry emails were sent to ensure if respondents received questionnaires.

### Data analysis

Mixed-method analysis was done for the data collected.

Quantitative data were analysed descriptively using Statistical Package for Social Sciences® (SPSS) version 21 and results presented as mean, median and percentages.

Burden of stroke care was estimated by dividing the number of stroke patients or patients with NCD having stroke as a complication over the total number of patients seen by the FMS in a period of one month. An estimation on burden of care and self reported practices was considered as the best approach as there has not been any local data available to estimate the proportion of stroke patients who receive treatment from the primary care physicians after discharge from tertiary care. Although generally most tertiary and secondary hospitals would discharge these patients to be followed up by the primary care team.

The data from the open-ended question were analysed using qualitative study principles. AFAA and NAMN were responsible for the analysis. AFAA transferred the written responses in its original form into a soft copy version. When responses were not legible or unclear, the respondents were contacted via email for further clarification. Both AFAA and NAMN viewed the data independently. Constant comparison method was used where ideas were continually checked with the original responses. The two researchers constantly read and re-read the responses, discussing themes and ideas until agreement was reached on defined themes. Respondents were recruited until data analysis showed saturation of themes. Quotations from respondents have been chosen on grounds of representativeness.

## Results

Of 121 respondents targeted, only 59 returned the questionnaire, making the response rate 48.8%.

### Background of the respondents

The mean age of the respondents was 40.5 (SD 7.5) years. The mean number of years in public service was 15.3 (SD 7.23) years, with an average of 7.55 (SD 4.16) years after postgraduate qualification i.e. MMed (Family Medicine). The majority of respondents were females (83.0%, 49/59) and ethnic Malays (72.9%).

The practice details are shown in Table 1. The calculated stroke care burden was 2 out of every 100 patients seen in the healthcentres.

**Table 1:** Practice details of FMS at Malaysian public healthcentres

	**Mean (SD), N (%)**			
Clinic sessions/week	4.60 (2.97)			
Number of primary care patients seen per session	19.32 (9.51)			
Number of stroke patients seen per month	7.17 (7.15)			
	Median 5 (IQR 2-10)			
Burden of stroke patients in primary care	0.02			
Geographical location of practice in Malaysia	**Peninsular**		**East Malaysia**	
	North	13	Sabah	1
	South	5	Sarawak	2
	East	5	Unspecified	3
	Central	30		
Place of practice	Rural		17	28.8%
	Urban		35	59.3%
	Rural & urban		7	11.9%
Estimated stroke patients seen at healthcentres/year	<20		32.2%	
	21-40		25.4%	
	41-60		6.8%	
	61-80		1.7%	
	81-100		3.4%	
	101-120		6.8%	
	Unsure		23.7%	

In terms of having a standard care plan for managing post stroke patients at primary care level, 72.4% (42/58) did not have one at their clinic, 19% (11/58) had a specific care plan while the remainder (8.6%, 5/58) were not sure if one existed. However, the majority 96.6% agreed that it was necessary to have a standardised care plan and this would improve quality of care for this group of patients.

Further analysis was done to determine if the location of practice would influence the current post stroke care provision was not statistically significant (Table 2).

**Table 2:** FMS opinion regarding post stroke care service provision based on practice/healthcentre location

**Post stroke care plans at health centres**	**Location of healthcentre**			**Fisher’s exact test**	**p**
	**Rural**	**Urban**	**Rural & Urban**		
Does your clinic/health centre have a standard care plan specifically for managing stroke patients?					
• Yes	2	9	0	2.282	0.272
• No	15	26	6		
Do you think it is necessary to have a standard care plan for you and your team for managing stroke patients?					
• Yes	16	34	6	1.053	1.00
• No	1	1	0		
In your opinion, do you think a standard care plan for stroke patients will help improve the quality of care for stroke patients in your health center?					
• Yes	16	34	6	1.053	1.00
• No	1	1	0		
Have you ever managed a patient who had features of acute stroke during a consultation?					
• Yes	12	30	4	2.594	0.293
• No	5	5	2		
Have you ever been consulted by your subordinates/staff on the management of patients who have had a stroke?					
• Yes	14	29	6	0.802	0.751
• No	3	6	0		
Overall, do you feel comfortable managing patients with stroke?					
• Yes	14	32	6	1.375	0.424
• No	3	3	1		

Table 3 lists the problems encountered by FMS in provision of care for post stroke patients at primary care level. The top 3 problems in general are related with transfer of care issues i.e. when the patient is discharged from the hospital and back to their own homes. On the other hand, it is encouraging to note that whilst reactive consultations to the primary care team does occur it is not highly ranked by the respondents.

**Table 3:** Obstacles encountered in provision of stroke care at primary level (1-most common, 10-less common)

^**#**^**Rank**	**N**	**%**	**Obstacle**
1	17/45	37.8	Lack of written information during transfer of care after discharge from hospital
2	14/52	26.9	Lack of information regarding rehabilitation therapy services
3	10/50	20.0	Lack of caregiver involvement
4	8/42	19.0	Most consultations are reactive
5	8/43	18.6	Delay in obtaining aids e.g. wheelchair
6	9/49	18.4	Unclear of available resources in the community & confusion regarding purpose of therapies
	7/38	18.4	
7	10/55	18.2	Poor overall/general care
8	7/44	15.9	Lack of self-interest and knowledge on stroke care management
9	8/51	15.7	Dealing with patient's emotional problems i.e. post stroke depression
10	6/42	14.3	Re-employment of patients with minimal neurological deficit

Table 4 demonstrates the type of referrals received and the type of services used by the Family Medicine Specialists in managing post stroke patients. Most of the services accessed are within Ministry of Health facilities. The three most common types of referrals being transfer of care type with no further follow-up by tertiary care team (88.1%), shared care between tertiary and primary care team (67.8%) followed by ‘in-house’ primary care patients developing stroke while under primary care management for NCD or other problems (64.4%). The top three ranking of services utilised in the management of post stroke patients while at primary care are Physiotherapy, Dietitian and followed by Speech & Language Pathologist.

**Table 4:** Types of referrals for post stroke management seen at primary care

**Referrals**	**N**	**%**
Patients referred for transfer of care from tertiary hospital to health centre (No further follow-up at Neurology or Physician Clinic)	52/59	88.1%
Patients referred for further monitoring in a shared care approach (i.e. with simultaneous follow-up at Neurology/Physician Clinic)	40/59	67.8%
Patients under FMS* care diagnosed as stroke	38/59	64.4%
Patients referred for further management by health care team (incl. nurses, Medical Assistants, Physiotherapist, Occupational Therapists etc.)	33/59	55.9%
Patients and/or carers requested for FMS intervention for stroke care	26/59	44.1%

*Family medicine specialists.

Table 5 lists the expectations of the FMS in improving the quality of care to post stroke patients. The findings, in order of importance reiterate the problems identified by the respondents in Table 3, which highlight the areas of inadequate instructions to primary care on care provision after discharge.

**Table 5:** Expectations for improvement of stroke care provision at primary care level (1-most common, 5-less common)

^#^**Rank**	**N**	**%**	**Expectations**
1st	37/55	67.3	Referral with adequate instructions and goals from discharging physician at tertiary or secondary hospital
2nd	27/54	50.0	Specific guidelines on management of long term stroke patients at community level
3rd	25/52	48.1	A web based system which prompts you on measures to be taken during follow-up of stroke patients at your centre
4th	23/56	41.1	Training programme or attachment at centres with expertise on community stroke care
5th	17/54	31.5	Regular meetings with Neurologists/Physician/Rehabilitation team to discuss specific problems of stroke patients managed at your centre

^#^1 = most common, 8 = least common.

### Knowledge on post stroke care provision

In this section, the median score for the 10 item-query on management of stroke risk factors was 8.0 (SD 1.6).

### Qualitative data

Altogether 53 respondents wrote their opinions with regard to aspects of stroke management which they perceive as being critical/important in primary care. Five themes emerged from the transcripts of the FMS response. The themes in priority list were; *the need for multidisciplinary team approach*, *access to rehabilitation services*, *patient and carer empowerment*, *the need for standardised care plan and availability of social support services*.

*The need for multidisciplinary team approach*: *Majority* of the respondents were of opinion that the management of post stroke patients in the community requires a multidisciplinary team approach (n = 20/53, 37.7%)

“(There should be a..) *Shared care* (plan) *with other teams e.g. physio* (physiotherapy), *occupational therapy*, *NGO*, *kebajikan* (welfare) etc. (R40)

“*Multidisciplinary*/ *team* (MDT) *management that can hasten the treatment as in primary care with many patient*(*s*) *and long waiting hours*, *care which is integrated on the system will compromised stroke patient* (waiting time). *MDT comprises of doctors*, *paramedics*/*community nurse for home visit*, *physiotherapist*, *dietician and welfare at least to serve stroke patient in a designated stroke clinic*…” (R46)

The respondents also emphasised the need for coordination of care between the tertiary centres and primary care, adopting a shared care approach.

“…(The) *Referral* (should come) *with adequate instruction*(*s*) *from tertiary hospital regarding long term goals* (which have been planned) *for the patient* (to achieve at community level)…” (R9)

“..(There should be better) *Support* (given) *by tertiary centres* (in providing expert advise on long-term-care goals and..) (*not dumping patient to the clinic*) (to manage as the primary care team chooses) ….” (R34)

*Access to rehabilitation services*: The respondents highlighted the need for availability of rehabilitation facilities to be based at primary care, i.e. healthcentres (n = 21/53, 39.6%).

Not all healthcentres in Malaysia have facilities for rehabilitation services [9], although there has been a recent move by the MOH to provide this facility at selected healthcentres with either physiotherapy alone or together with occupational therapy services. In some healthcentres, a visiting physiotherapist or occupational therapist provide services on rotational basis for healthcentres. However, the rehabilitation service at healthcentres is not solely for stroke rehabilitation. Therapists also have to cater for other services such as acute pain service, rehabilitation for amputees for example [1].

“(There is) *Problem of rehabilitation care as our patient mostly having difficulty to go to rehab centre which is at the tertiary hospital*. (There is a …) *Need of physiotherapist & occupational therapist* (to be based) *in health centre* (as rehabilitation) *is most critical in stroke care*.” (*R23*)

“(There should be) *More* (accessible secondary care) *service* –*physiotherapy*, *OT* (Occupational Therapist) (to be based) *in primary care clinic*” (*R38*)

The above findings are similarly highlighted in the ranked list of rehabilitation services used by the FMS in provision of post stroke care in Table 6.

**Table 6:** Rehabilitation services used in management of post stroke patients in last one year

^#^**Rank**	**N**	**%**	**Rehabilitation service**
1st	23/59	39.98	Physiotherapy (MOH*)
2nd	7/59	11.86	Dietitian
3rd	4/59	6.78	Occupational therapy (MOH)
4th	7/59	11.86	Social welfare
5th	7/59	11/86	Speech & Language Therapy (MOH)
6th	5/59	8.47	Prosthetics & Orthotics (MOH)
7th	4/59	6.78	NGO
8th	8/59	13.56	Occupational therapy (Private)

^#^1 = most common, 8 = least common.

*Ministry of Health, Malaysia.

*Patient and carer empowerment*: Apart from type of care and service-related issues, the FMS acknowledged the impact of patients and their caregivers’ efforts and motivation to obtain optimal post discharge care for stroke. Another apparent theme observed was the impact of the patients’ and caregivers’ effort after discharge to the patient’s overall well-being. The level of commitment from both patients and caregivers was singled out as an important feature to ensure successful post stroke care delivery (n = 17/53, 13.2%).

“*To educate and empower patient and carer regarding home management of stroke*; *short and long term outcome of stroke and risk of recurrent stroke*.” (*R1*)

“(The most important aspect for post stroke management at primary care is the..) *Commitment from patients & relatives*/*family*.” (*R34*)

*The need for standardised care plan*: The respondents indicated there was a need for a standard care plan or guideline that addressed components of stroke management at community level (i.e. further rehabilitation, management of stroke risk factors and re-integration into community) (n = 7/53, 13.2%)

“(There should be a) *Well*-*written* (*care*) *plan* (to guide the primary care team)” (*R11*)

“*Should have* (a) *standard care plan for stroke management at health clinic level*.” (*R16*)

“*Knowledge among the health care provider and the community* (regarding post stroke monitoring and rehabilitation) *needs to be strengthened*.” (*R29*)

Respondents were familiar with the management of stroke risk factors. However, these conditions were generally managed as NCD patients per se, at the expense of other post stroke complications such as need for further rehabilitation, screening for depression and/or vascular dementia for example. Specifically, management of these complications was difficult particularly for the FMS who have not had training in rehabilitation or elderly care for instance.

*The availability of social support services*: The importance of social support services is included as a vital aspect of managing post stroke patients. Social welfare workers should be included in the multidisciplinary team caring for post stroke patients in the community (7/53, 13.2%).

“(The) *Social worker need to be actively involved in the care of not only the patient but also* (the) *carers* (caregivers)/*involved family members*.” (*R29*)

## Discussion

There is scarcity of data, which addresses the Family Medicine Specialists or general practitioners’ views on provision of care for stroke patients in the Malaysian community. In a setting where care guidelines are lacking and the transfer of care from tertiary to primary care is fragmented for post stroke patients, we attempt to identify the gap where current public healthcare delivery can be improved upon. The quantitative portion of this study complements the qualitative data obtained.

The low response rate among the FMS suggests a possibility of low awareness on the provision of care for stroke patients at public healthcentres in the community. It is highly likely that post stroke patients continue to receive treatment for stroke risk factors (i.e. hypertension, diabetes, obesity) and managed as chronic non-communicable disease protocol, which minimally addresses stroke rehabilitation.

Based on the respondents’ estimation, the burden of care for stroke patients is low, with only 2 out of every 100 patients seen at primary care being stroke patients. Considering the number of strokes occurring is estimated at 40,000 per year in Malaysia (NASAM) [10], and the increasing number of stroke survivors due to decline in stroke mortality since the past five years, this suggests a low proportion of post stroke patients are seen at public primary care facilities. Hence, this may indicate a possible problem occurring at point of transfer of care from hospital into the community. On the other hand, the low utilisation of public primary care facilities could possibly be due to patients who continue post stroke follow-up at Specialist Stroke care centres in the public or private hospitals and clinics. The longer opening hours, shorter waiting time and services which run beyond office hours and also weekends at private general practices are some caregiver-friendly reasons which may influence post stroke patients and their caregivers to continue post stroke monitoring at private healthcare facilities. The private general practices in this country however, consist of both trained and not-formally trained primary care practitioners. As such, added with the scarcity of local data, which estimates the proportion of patients who receive post stroke treatment from which type of health facility after the acute period makes it difficult to account on how efficient post stroke care is delivered.

Further reasons on why few stroke patients are managed at primary care level should be explored. The lack of stroke registries at health centres complicates actual assessment of the burden of care for post stroke patients at primary care level. The setting up of the National Acute Stroke registry in 2009 is therefore, timely. We feel that with the addition of the stroke registries at local healthcentres, a strong database will be established as per WHO recommendation for developing countries i.e. The Steps Stroke Surveillance System. A complete database encompasses step1-events in the hospital, step2-related to fatal events in the community and step3-related to nonfatal events in the community [8,11]. The use of registries in developed nations to assess the impact of organised stroke services has helped the stakeholders to plan and monitor services with the aim to reduce burden of care [12,13]. The current Acute Stroke Registry, which is based at tertiary hospitals, has limited information on patients who do not continue post stroke follow up at these hospitals.

In our study, coordination of care for patients was identified as an issue. Mitchell and colleagues in their review described the models of care coordination of stroke patients involving the primary care team [14]. The secondary outreach model appears to be the most common used. Two variants of this model were described. In one model, transfer of care and responsibility of post stroke patients became the responsibility of the primary care team, which is supported by community hospital based team [15]. This model also catered for patients with stroke whom never left the care of the GP and were never admitted to hospital during the acute stroke [16,17].

The other model was a Specialist-led service, which involved nurses as care coordinators in the community. Nurses followed up patients who were discharged [16]. Allied health personnel based in the same unit or with the local primary care provider aided these nurses. The effectiveness of models of care however has not been proven. The current system in Malaysia and most likely in developing countries is that primary care teams are unlikely to get as much support from the limited and overwhelmed Specialist Stroke services as per the latter model. Specialist stroke services are largely based at tertiary centres in urban areas. Hence, the model, which best suits, this setting would be to depend on FMS-led post stroke service which utilises healthcentres (community based) allied health services. To date; FMS-led primary care teams are based all over the country in urban, suburban and rural areas of Malaysia. At the time the study was conducted, the FMS (in public service) to population ratio was 1:153409. This is almost 8 times above and beyond the responsibility of providing care for districts per FMS at health centre with 15-20,000 populations. However, with the aid of (approximately 7000) primary care physicians in private practice, the ratio is further reduced to 1:3991, thus making provision of primary care led post stroke care delivery a possibility worth considering.

In our local setting, the majority of patients discharged from tertiary care after stroke episodes were referred to FMS and/or primary care team at the public healthcentres for further management. Inadequate instructions and goals for long term management for the patient were not relayed to the primary care team. Apart from managing stroke risk factors, the FMS had limited feedback or guidance on rehabilitation goals for the post stroke patient. The FMS based at the public healthcentres fulfills the role of a Clinical Specialist providing guidance and expert advise to the primary care team at the public healthcentres. We hope that by providing the primary care teams with care protocols developed by a panel of local Specialist Stroke care providers and tailoring it to the local health services structure will provide a link for the gap which currently exists in this country and probably other developing countries. Reducing inequity of care is the top priority when other components of multidisciplinary rehabilitation team are not accessible [18-21].

The multidisciplinary care team approach that was mentioned by the respondents mainly identified resources, which were available in the local setting. Identified team members apart from the FMS, were the Physiotherapists and Occupational Therapists, Social workers and paramedics. This differed slightly from the line-up given from literature from developed public health systems – which comprised of a specialist physician, the patient’s GP, care coordinators or nurse, physiotherapist, occupational therapist with others (like speech therapists or administrative staff) being included in the teams [14,22]. The suggested team members are an option to consider for certain tasks, which can be delegated to cope with limitations in staffing at healthcentres. With adequate training and supervision, tasks like maintaining a community stroke registry may be handled by a therapist or a trained paramedic can do assessment of cognitive function.

In some models of care, decisions are made and enacted by the specialist team [16,23]. In other models, there is deliberate decision to transfer care and responsibility to primary care providers. This was done by supported discharge [24], transferring patients to a GP-led community hospital based team [15] or to provide services to patients with stroke who never left the care of the GP, and did not get admitted to hospital in the acute phase [16,17]. Acknowledging the shortage of Specialist Stroke services, e.g. Neurologists and Internal Physicians with interest in Stroke care provision as well as Rehabilitation Physicians in this country, a coordinated, shared care approach with FMS’ is most beneficial. FMS’ based in public healthcentres in urban and rural parts of Malaysia would be the link to provide a ‘guided-primary-care driven’ post stroke care service for patients residing in the community.

Most literature on stroke care in developed countries has showed effectiveness of early supported discharge after an acute stroke and its benefits on patient outcomes [24-28]. In these countries, established facilities such as domestic rehabilitation services and community rehabilitation centres or hospitals and assisted living services are available to assist patients in their recovery following hospital discharge. However, in the majority of developing countries, the scenario of ‘early discharge, minimal or no support’ is generally the norm. Most Specialist stroke care services are located in urban-based tertiary hospitals. After the average stay of about 5-7 days [6,29], patients are discharged to be cared by relatives in their own homes. Rehabilitation continues mostly as an outpatient or daycare service at tertiary or secondary care hospitals. Patients return to the hospital for rehabilitation sessions, which mostly depend on availability of resources as well as patients’ (and their caregivers’) capability to come to the hospital for the therapy sessions. Most patients and their caregivers tend to default the sessions due to time and financial constraints and possibly lack of awareness on benefits of post stroke rehabilitation. Based on these factors, the respondents saw the need to equip the healthcentres with rehabilitation facilities to improve accessibility for patients who still require rehabilitation. The need for community based facilities for stroke rehabilitation is also stressed upon by the respondents to ensure patients achieve maximal potential for functional recovery. This is also supported by the ranked list of facilities most likely referred in the course of providing post stroke care. From the study, we note that the problem of access to rehabilitation was a problem among urban-based health centres. As such, it is expected that healthcentres in rural settings would face greater challenges in providing rehabilitation to post stroke patients.

While there were attempts to improve access to rehabilitation services at local healthcentres in the last few years, guidelines for provision of long-term rehabilitation are lacking [9]. Selected healthcentres in the country have Physiotherapy and/or Occupational Therapy services, on site or on rotation basis. Although largely responsible for provision general rehabilitation services and specialised services (e.g. Children with special needs or Elderly rehabilitation services), services for post stroke rehabilitation is limited. The lack in agreement of definition of long term period [30], lack in local guidelines for post stroke rehabilitation at healthcentres are some of the issues which need to be addressed to provide better access to post stroke patients in the community.

Having a one-stop centre which is able to provide both risk factor monitoring as well as rehabilitation would ideally benefit both patient and their carer in reducing the time and money spent on going to different venues for post stroke care.

The majority of respondents were senior and experienced family physicians that had been serving public healthcentres for an average of 7.5 years after postgraduate training. Along with the geographical location of the FMS respondents, which represented all the states in Malaysia, their views on the provision of post stroke care in this country are quite relevant. The location of the pratice was not associated with the current practice of post stroke care provision, suggesting that the problem was inherent and warrants remedial intervention. In terms of assessing perception of the respondents, there was adequate representation and saturation of themes, which was complemented by the quantitative portion of this study.

## Conclusions

It is acknowledged that post stroke care at community level is fragmented even in most well- established public health systems globally. For Malaysia and most developing countries in the region, the challenge is greater in trying to provide a seamless transfer of care beyond the acute phase. The lack of coordinated care has resulted in patients and their carers turning to primary care providers for various stroke related problems as well as other non-stroke related issues.

Support and guidance for primary care team from Specialist stroke care providers to coordinate and deliver post stroke care is needed locally. A locally developed care pathway or case management protocol which utilises local healthcare resources and catered to overcome the inadequacies in the local public health system would be most beneficial for both the patients as well as Stroke care providers. Concerted efforts to link tertiary and secondary care with primary care is essential to ensure patients will receive optimal care following discharge from the acute care. Seamless transition of care is vital to enable patients to not only receive stroke risk factor monitoring but also commence or continue further rehabilitation as well as allow community re-integration. There is a need to progress from the current ‘early discharge without support’ scenario to the proven effective ‘early supported discharge’ type of care. Provision of rehabilitation services at community healthcentres facilities will enable further rehabilitation to take place for patients who have been discharged from tertiary care. For stroke patients who have been under the care of their FMS or GPs or ‘prevalent stroke’ in the community, access to rehabilitation services should improve if post stroke care was given in the same premise.

This information has allowed us to proceed with the development of an integrated care pathway for post stroke patients who have been discharged to the community as well as patients who have had stroke but remained under the care of the FMS and/or primary care team. The main objective for the pathway would be to provide coordinated and optimal post stroke care for patients in the community in areas with limited access to Stroke Specialist services.

## Abbreviations

FMS: Family medicine specialists; NASAM: National stroke association of Malaysia; NSPNCD: National strategic plan for the management of non communicable diseases.

## Competing interests

The authors declare that they have no competing interests.

## Authors’ contributions

AFAA conceived of the study, participated in its design and coordination and drafted the manuscript. SA participated in the study design and helped coordinate the survey. AFAA & NAMN performed the analysis of both quantitative and qualitative data. NAA, SS and SMA provided expert content guidance. All authors read and approved the final manuscript.

## Authors’ information

AFAA is a senior lecturer and Family Medicine Consultant at Faculty of Medicine, Universiti Kebangsaan Malaysia Medical Centre. NAMN is a senior lecturer and physiotherapist at School of Rehabilitation Sciences, Faculty of Health Sciences, Universiti Kebangsaan Malaysia. Both are doctoral candidates at the United Nations University International Institute for Global Health in Kuala Lumpur. NAA is a Family Medicine Consultant and Stroke Rehabilitation Specialist at the Faculty of Medicine, Universiti Kebangsaan Malaysia Medical Centre. SA is a Family Medicine Specialist at Klinik Kesihatan Marang, Terengganu and Webmaster for the Malaysian Family Medicine Specialist Association website. SS is a Public Health consultant and Head of Department of Health Information, Universiti Kebangsaan Malaysia Medical Centre, Kuala Lumpur, Malaysia and; SMA is a Public Health Consultant, Professor of Health Economics and Senior Research Fellow, United Nations University-International Institute for Global Health, Kuala Lumpur, Malaysia & Head of the International Casemix & Clinical Coding Centre in Kuala Lumpur.

## Pre-publication history

The pre-publication history for this paper can be accessed here:

http://www.biomedcentral.com/1471-2296/15/40/prepub
